# Does Poverty Explain All? A Path Analysis of Single Mothers’ Dietary Behaviors in Japan

**DOI:** 10.3390/nu18101509

**Published:** 2026-05-09

**Authors:** Lin Wu, Akira Ishida

**Affiliations:** Graduate School of Agricultural Science, Kobe University, Kobe 657-8501, Japan

**Keywords:** single motherhood, maternal diet, economic inequality, intergenerational co-residence, mediation analysis, Japan

## Abstract

**Background/Objectives**: In Japan, single mothers face a high risk of poverty and poorer dietary quality than partnered mothers; however, the pathways underlying these disparities remain unclear. This study examined whether economic status mediates the association between family structure and maternal dietary behaviors and whether intergenerational co-residence modifies these pathways. **Methods**: Using nationally representative repeated cross-sectional survey data from 2018 to 2022 (*N* = 1319 mothers), we conducted path analysis of dietary awareness and behaviors. Family structure was classified as partnered mothers (reference), single mothers not co-residing with grandparents, and single mothers co-residing with grandparents. Economic status and frequency of shared family dinners were modeled as mediators. **Results**: Compared with partnered mothers, both groups of single mothers showed poorer economic conditions, with greater disadvantage among those not co-residing with grandparents. Economic conditions accounted for substantial disparities in multiple dietary outcomes. Shared family dinners were positively associated with several dietary behaviors; however, dinner frequency did not differ significantly between partnered and single mothers or between single mothers with and without grandparental co-residence. Single mothers not co-residing with grandparents showed residual direct disadvantages in nutritionally balanced meals, breakfast frequency, and healthy dietary awareness, whereas those co-residing with grandparents showed weaker disadvantages. **Conclusions**: Dietary vulnerability among single mothers in Japan was strongly associated with economic disadvantage, but not poverty alone. Intergenerational co-residence was associated with weaker disadvantage rather than eliminating it. Policies should combine income support with childcare assistance and low-burden *Shokuiku* or community-based meal support to strengthen resource capacity and daily healthy eating routines.

## 1. Introduction

Maternal dietary consciousness and behaviors are key determinants of the nutritional well-being of mothers, their children, and the household. Evidence indicates that mothers’ eating habits and feeding practices shape children’s dietary awareness, food preferences, and long-term health trajectories that persist into adulthood, highlighting the intergenerational impact of maternal diet [1,2,3,4]. In Japan, however, maternal dietary practices have become increasingly unstable due to unpredictable external shocks. National studies reported deterioration in dietary consciousness during the COVID-19 pandemic [5,6,7], as well as substantial adjustments in food consumption in response to rising food prices [8]. These changes have occurred alongside rapid transformations in family structure, including the decline of three-generation households and the growth of dual-earner and single-parent families [9,10]. Among these groups, single-mother households represent the most vulnerable population. As of 2016, 86.8% of the 1.23 million single-parent households were headed by mothers, more than half were living in poverty, and only about 4% received public assistance [11]. Although single mothers often demonstrate strong concern for their children’s diets, their households are more likely to exhibit irregular meals and other unhealthy eating patterns [12,13]. Given their increasing prevalence and central role in shaping children’s dietary development, identifying the mechanisms underlying single mothers’ dietary behaviors is essential for sustaining long-term nutritional health in Japan.

Previous studies on dietary behavior can be broadly divided into two streams. The first has focused on socioeconomic status, demonstrating the effects of income, employment status, education, age, marital status, and region on dietary quality [14,15,16,17,18]. The second has emphasized physiological and psychological factors, including loneliness, stress, and lifestyle behaviors [19,20]. Research on single mothers has largely followed a comparative approach, documenting poorer dietary outcomes for both mothers and children relative to those in two-parent households [12,13]. However, these studies have typically treated single mothers as a homogeneous group and modeled family structure and economic status as parallel predictors rather than as components of a causal mechanism. Although shared family meals are consistently associated with better diet quality and psychosocial outcomes [21,22], the role of intergenerational co-residence within single-mother households remains largely unexplored. This gap represents a crucial limitation because, from the perspective of household production theory, family structure is not merely a demographic characteristic but a system that organizes the allocation of time, labor, and material resources [23,24].

Single motherhood is widely recognized as a structural condition of socioeconomic disadvantage. Single mothers are more likely to experience a higher risk of poverty, unstable employment, time scarcity due to the dual burden of paid work and childcare [25,26], and elevated psychological stress [27,28,29]. These factors have been linked to irregular eating patterns, lower diet quality, and greater reliance on inexpensive, energy-dense foods [15,18,20,25,26,30,31]. Moreover, financial strain and caregiving fatigue can negatively affect the mealtime environment and reduce opportunities for shared meals [21], which are important for establishing healthy dietary routines for children [22]. However, despite the robust associations documented in the literature, two key questions remain unresolved: whether economic disadvantage functions as a mediating pathway linking single motherhood to dietary outcomes and whether single mothers constitute a homogeneous group in terms of available family resources.

Intergenerational co-residence may fundamentally reshape the constraints faced by single mothers. From the perspective of household production theory and family resource distribution, living with grandparents can influence dietary behaviors through multiple pathways. First, it may provide economic support through income pooling and the sharing of housing and food expenditures. Second, it may alleviate time constraints by providing childcare and assistance with meal preparation, thereby increasing the capacity for home cooking and regular meals. Third, it may buffer psychological stress by offering emotional support and stabilizing the household environment. Previous studies in Japan have shown that intergenerational households are associated with greater food sharing and higher levels of psychological well-being, suggesting a potential protective mechanism for dietary health [32,33].

Within this framework, economic status and the frequency of family dinners can be conceptualized as key mediating pathways linking family composition to dietary behaviors. Economic conditions determine purchasing power, food affordability, and the ability to secure nutritionally balanced meals. At the same time, shared dinners represent an observable behavioral indicator of available time resources. Rather than measuring time constraints as an abstract construct, the frequency of eating dinner with family members directly reflects whether time availability is translated into concrete dietary practices. If intergenerational co-residence reduces the time burden on single mothers, this reduction should be reflected in a higher likelihood of shared meals, which in turn is associated with healthier eating patterns. Modeling these variables as mediators therefore provides a more explicit and behaviorally meaningful test of the underlying mechanisms.

Despite growing attention to single-mother households in Japan, three critical research gaps remain. First, single mothers have largely been treated as a homogeneous population without distinguishing differences in intergenerational living arrangements. Second, the role of co-residence with grandparents in shaping dietary behaviors has rarely been examined quantitatively. Third, the mediating mechanism through which economic status links family structure to dietary outcomes has not been empirically tested using structural modeling approaches.

To address these gaps, this study adopts a structural perspective to examine how family composition and economic status jointly shape maternal dietary behaviors. Using five waves of nationally representative cross-sectional data collected from 2018 to 2022, we apply path analysis to test the mediating role of economic conditions and the differentiating role of intergenerational co-residence. Specifically, this study aims to (1) distinguish single mothers by co-residential status, (2) examine disparities in economic conditions across different family types, and (3) test whether economic status mediates the relationship between single motherhood and dietary behaviors.

By clarifying the mechanisms underlying dietary disadvantage, this study contributes to the literature in three important ways. First, it moves beyond descriptive comparisons and provides a structural explanation of how family composition influences maternal dietary behaviors through material pathways. Second, it introduces intergenerational co-residence as a key source of heterogeneity within single-mother households. Third, it provides policy-relevant evidence for designing food education and family support programs that target the most vulnerable groups—particularly single mothers who lack intergenerational support. Improving maternal dietary practices in such households is essential not only for mothers’ health but also for the nutritional development of the next generation.

## 2. Materials and Methods

### 2.1. Sample

This study utilized data from five independent waves of the Survey of Attitudes toward Food and Nutrition Education (*Shokuiku* in Japanese), conducted annually from October to December by the Ministry of Agriculture, Forestry and Fisheries of Japan between 2018 and 2022. The Basic Act on *Shokuiku* requires the government to submit an annual report to the Diet on measures taken to promote *Shokuiku*, for which these surveys provide background information. Each wave is a nationally representative repeated cross-sectional survey targeting individuals aged ≥20 years residing in municipalities nationwide and employs a stratified two-stage sampling design. Survey administration differed across periods. In 2018–2019, the survey was conducted through in-person individual interviews by trained interviewers. In 2020–2022, data collection shifted to self-administered questionnaires using mail and web-based modes. From the full dataset, observations with complete data on all study variables were retained. Among these, 1319 mothers living with children under 18 years of age were included in the analysis, of whom 110 were single mothers.

### 2.2. Dietary Behaviors and Awareness

The *Shokuiku* Awareness Survey collects information on dietary awareness, eating behaviors, and household socioeconomic conditions. For the purpose of this study, five ordinal outcome variables representing dietary behaviors were selected: (1) frequency of nutritionally balanced meals including staple foods, side dishes, and main dishes; (2) frequency of eating breakfast; (3) attention to healthy dietary practices; (4) dietary practices aimed at preventing lifestyle-related diseases, such as maintaining an appropriate body weight and limiting salt and fat intake; and (5) the practice of eating slowly and chewing well. In the official survey materials, staple foods refer to grain-based items such as rice, bread, and noodles that primarily provide carbohydrates and energy; main dishes refer to dishes centered on fish, meat, eggs, or soy products that mainly supply protein and fat; and side dishes refer primarily to vegetable-based dishes that complement the meal with vitamins, minerals, and dietary fiber. The corresponding survey item asks respondents how many days in a usual week they consume this combination at least twice per day, thereby capturing repeated daily meal balance rather than occasional balanced eating.

All outcome variables were measured using four-point Likert scales and treated as ordinal variables in the analysis. The first two outcomes were coded as 1 = very low, 2 = low, 3 = high, and 4 = very high. The latter three outcomes were coded as 1 = less than one day/week, 2 = 2–3 days/week, 3 = 4–5 days/week, and 4 = 6–7 days/week. Higher values indicate healthier or more favorable awareness and behaviors.

### 2.3. Single Parenthood and Mediators

Family structure was operationalized into three mutually exclusive groups: single mothers not co-residing with grandparents (SingleP1), single mothers co-residing with grandparents (SingleP2), and partnered mothers (married or partnered mothers living with their spouse and children), which served as the reference group. SingleP1 and SingleP2 were treated as binary variables with partnered mothers as the reference category. This classification enables direct comparisons between single-mother and partnered-mother households while distinguishing the potential buffering role of intergenerational co-residence. The key mediator was economic status, measured by the self-reported perceived household financial situation on a five-point Likert scale and reverse-coded so that higher values indicate greater financial affluence (1 = not affluent at all to 5 = very affluent). Objective household income data were not available in the *Shokuiku* survey. Therefore, perceived economic status was used as the primary indicator of economic status, as subjective economic strain is closely related to everyday food purchasing constraints and diet-related decision-making and has been linked to unhealthy dietary habits in prior studies [34,35]. In addition, we included the frequency of shared family dinners (five-point Likert scale, ranging from 1 = rarely to 5 = almost every day) as a potential mediator capturing household routines and time constraints. Time affluence (five-point Likert scale, coded in the same direction as economic status) was initially considered as an additional mediator and evaluated through model comparisons.

### 2.4. Other Covariates

Control variables included mothers’ age, residential type (ordinance-designated city vs. other municipalities), and survey-year dummy variables (2020, 2021, and 2022) to capture temporal, urban residential-context, and pandemic-related influences. An ordinance-designated city refers to a city with a population of 500,000 or more, designated by Cabinet Order under the Local Autonomy Law. These cities have administrative authority comparable to that of prefectures and provide services to residents through their own administrative districts. There are currently 20 ordinance-designated cities in Japan, including Sapporo, Kyoto, Osaka, and Kobe. This variable was included as a coarse indicator of urban residential context, reflecting broad differences in household economic conditions and local policy environments, including variation in municipally implemented child-rearing and food-related support.

### 2.5. Data Analysis

We conducted a path analysis to examine the mediating role of economic status in the association between family structure and a series of dietary behaviors while controlling for other covariates, including mothers’ age, residence, and survey year. Because the five dietary outcomes were ordinal, models were estimated using the *lavaan* package in R (version 4.5.2) with an estimator appropriate for ordered categorical outcomes (WLSMV), and results were reported as standardized coefficients.

To assess the contribution of each mediator, we compared a full model including economic status, time affluence, and shared dinner frequency with nested models that removed one mediator at a time, using scaled χ^2^ difference tests for model comparison. Based on these comparisons, the final model specification prioritized parsimony and interpretability while retaining mediators that materially contributed to model fit. Time affluence was moderately correlated with economic status (r = 0.40) and its removal did not significantly worsen model fit; therefore, it was omitted from the final model. Correlation and multicollinearity diagnostics for variables included in the path models were examined and are reported in the Appendix A (Table A1). The lavaan model syntax is available from the authors upon reasonable request.

Several hypotheses were derived from previous findings. First, single motherhood was expected to be associated with unfavorable dietary awareness and practices, based on prior evidence documenting the vulnerability of single mothers with respect to dietary conditions. Second, higher economic status, older age, and residence in an ordinance-designated city were expected to be associated with more favorable dietary behaviors, because greater financial resources and fewer external constraints may expand dietary options and improve diet quality. Third, economic status was hypothesized to mediate the association between single motherhood and dietary outcomes. Fourth, co-residence with parents was expected to offset the negative effects of single motherhood on dietary outcomes through economic status, as grandparents would likely provide financial and child-rearing support that improves overall well-being. Due to the absence of validated measures of stress or psychological distress in the survey, the stress–buffering pathway could not be directly modeled; instead, self-rated health and related behaviors were treated as downstream outcomes, and the stress mechanism is discussed in the Discussion and Limitations sections.

## 3. Results

### 3.1. Respondents’ Characteristics

The analytic sample comprised 1319 mothers. The mean age (standard deviation [SD]) was 41.37 (±6.88) years. The mean economic status score (five-point scale) was 3.13 (SD = 0.99), and the mean time affluence score (five-point scale) was 2.72 (SD = 1.12) (see Table 1). Regarding family structure, 2.88% (*n* = 38) were single mothers living with grandparents (SingleP2), 5.46% (*n* = 72) were single mothers not living with grandparents (SingleP1), and 91.66% (*n* = 1209) were partnered mothers. For dinner co-eating frequency, 81.58% (*n* = 1076) reported “almost every day,” 8.04% (*n* = 106) reported “4–5 days/week,” 7.05% (*n* = 93) reported “2–3 days/week,” 1.90% (*n* = 25) reported “1 day/week,” and 1.44% (*n* = 19) reported “rarely.” A total of 30.17% (*n* = 398) lived in an ordinance-designated city. The survey-year distribution was as follows: 2018, 18.88% (*n* = 249); 2019, 17.44% (*n* = 230); 2020, 20.70% (*n* = 273); 2021, 22.14% (*n* = 292); and 2022, 20.85% (*n* = 275).

### 3.2. Model Comparisons and Regression Results

Four path models were compared: one including three mediators (economic status, time availability, and frequency of shared family dinners) and covariates including age, region, and survey year indicators for 2020, 2021 and 2022 relative to 2018 and 2019, and three additional models, each removing one mediator. The full path model demonstrated good fit: χ^2^ = 7.06, df = 5, CFI = 0.999, TLI = 0.992, RMSEA = 0.018, and SRMR = 0.001 (Table 2). In nested model comparisons based on the scaled χ^2^ difference test (vs. the full model), removing time affluence did not significantly change model fit (Δχ^2^ = 5.32, Δdf = 5, *p* = 0.378). In contrast, removing shared dinner frequency significantly worsened model fit (Δχ^2^ = 46.0, Δdf = 5, *p* < 0.001), and removing economic status also significantly worsened model fit (Δχ^2^ = 67.2, Δdf = 5, *p* < 0.001). Therefore, the standardized results from the model excluding time affluence are presented in Table 3 to enhance parsimony and interpretability. The final path model demonstrated good fit: χ^2^ = 7.23, df = 5, CFI = 0.998, TLI = 0.992, RMSEA = 0.018, and SRMR = 0.001. Correlation and multicollinearity diagnostics for all variables included in the path models are reported in the Appendix A (Table A1).

Table 3 summarizes the standardized associations for five dietary behaviors (dietary balance, breakfast consumption, healthy awareness, healthy dietary practices, and slow eating), distinguishing mediated pathways through economic status and shared dinner frequency from the direct associations of other covariates. Across outcomes, economic status was positively associated with dietary balance (β = 0.11), healthy awareness (β = 0.20), healthy dietary practices (β = 0.19), and slow eating (β = 0.14; all *p* < 0.001), but not with breakfast consumption. In contrast, shared dinner frequency was positively associated with dietary balance (β = 0.14), breakfast consumption (β = 0.18), healthy awareness (β = 0.19), and slow eating (β = 0.12; all *p* ≤ 0.003), but not with healthy dietary practices. Beyond these two mediating pathways, several direct associations were observed. SingleP1 (single mothers not living with grandparents) showed negative direct associations with dietary balance (β = −0.13), breakfast consumption (β = −0.15), and healthy awareness (β = −0.09; all *p* < 0.01), whereas SingleP2 (single mothers living with grandparents) showed no statistically significant direct associations with the five dietary behaviors in the final model. Age was positively associated with dietary balance (β = 0.10), healthy awareness (β = 0.10), and healthy dietary practices (β = 0.11; all *p* < 0.01). Survey-year indicators (2020–2022) were negatively associated with dietary balance and slow eating.

**Figure 1:**
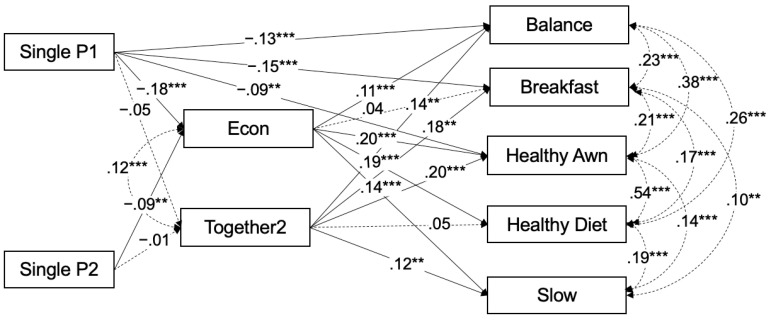
Path diagram summarizing the main pathways linking family structure, mediators, and dietary behaviors. Notes: Standardized coefficients are shown. Solid arrows indicate statistically significant paths, and dashed arrows indicate non-significant paths. The dashed double-headed arrow indicates the covariance between mediators. Models are adjusted for age and survey-year indicators in the dietary outcome equations, and additionally for region (ordinance-designated city) in the mediator equations. Non-significant paths from single parenthood to dietary behaviors are omitted for clarity. ** *p* < 0.01, *** *p* < 0.001.

Determinants differed between the two mediators. For economic status, both single-mother groups were negatively associated with economic status (with a larger magnitude for SingleP1 than for SingleP2); residence in an ordinance-designated city was positively associated with economic status (β = 0.07, *p* = 0.013), and later survey years (2021–2022) were negatively associated with economic status, whereas age was not statistically significant. For shared dinner frequency, neither SingleP1 nor SingleP2 showed a statistically significant association. Instead, shared dinner frequency was negatively associated with age (β = −0.16, *p* < 0.001) and positively associated with survey-year indicators (2020–2022). Overall, Table 3 indicates that family-structure differences are expressed primarily through economic status and selected direct associations, whereas shared dinner frequency varies mainly by age and survey period. The pathway contrast by single-mother subgroup is illustrated in Figure 1.

### 3.3. Path Diagram

Figure 1 illustrates the pathways linking family structure to dietary behaviors, contrasting single mothers not living with grandparents (SingleP1) with those living with grandparents (SingleP2). Relative to partnered mothers, both single-mother groups were negatively associated with economic status (SingleP1: β = −0.18, *p* < 0.001; SingleP2: β = −0.09, *p* < 0.001), with a larger magnitude for SingleP1. Neither SingleP1 nor SingleP2 was significantly associated with shared dinner frequency (SingleP1: β = −0.05, *p* = 0.142; SingleP2: β = −0.01, *p* = 0.702). SingleP1 also showed direct associations with less frequent nutritionally balanced meals, breakfast consumption, and attention to healthy dietary practices. Economic status (econ) was positively associated with nutritionally balanced meals, attention to healthy dietary practices, healthy dietary practices aimed at preventing lifestyle-related diseases, and slow eating, whereas shared dinner frequency (together2) was positively associated with all dietary outcomes except healthy dietary practices aimed at preventing lifestyle-related diseases. Figure 1 further shows a positive correlation between economic status and shared dinner frequency (β = 0.12, *p* < 0.001), as well as positive correlations among the five dietary outcomes.

### 3.4. Direct, Indirect, and Total Effects

Table 4 clarifies the extent to which economic disadvantage accounted for dietary vulnerability among single mothers by decomposing total effects into direct effects and indirect effects via economic status. For SingleP1, significant negative total effects were observed for dietary balance, breakfast consumption, healthy dietary awareness, and healthy dietary practices, indicating the clearest overall disadvantage relative to partnered mothers. Indirect effects via economic status were significant for dietary balance, healthy dietary awareness, and healthy dietary practices, but not for breakfast consumption, whereas direct effects remained significant for dietary balance, awareness, and breakfast consumption. These findings indicate that economic disadvantage explained part, but not all, of the SingleP1 disadvantage. For SingleP2, significant indirect effects via economic status were observed for several outcomes; however, only healthy dietary awareness retained a significant negative total effect. Thus, the decomposition suggests that economic disadvantage accounted for a broader and more substantial share of dietary vulnerability among SingleP1, whereas among SingleP2 its explanatory role was more limited and did not translate into similarly consistent overall disadvantage across outcomes.

## 4. Discussion

The present study examined whether economic disadvantage and time-related constraints explain dietary vulnerability among single mothers in Japan and whether intergenerational co-residence differentiates these pathways. The core finding is that SingleP1 constituted the most vulnerable group, showing the clearest disadvantages in nutritionally balanced meals, breakfast consumption, and health-oriented dietary awareness. These disadvantages were expressed mainly through the economic pathway and a limited set of residual direct associations, whereas SingleP2 showed weaker overall disadvantages. This pattern indicates that dietary vulnerability is structurally patterned by household resource capacity and living arrangements rather than reflecting a uniform “single-mother effect.” Overall, economic disadvantage accounted for a substantial share of dietary vulnerability, but it did not fully account for the disadvantages observed among single mothers, particularly SingleP1.

The mediation results clarify how family structure is associated with dietary awareness and behaviors. First, economic status emerged as the dominant mechanism: both single-mother groups were associated with poorer perceived economic status (with a stronger association for SingleP1), and economic status, in turn, was positively related to multiple dietary outcomes (dietary balance, healthy awareness, lifestyle-related disease prevention practices, and slow eating). This pattern supports socioeconomic explanations of dietary inequality (e.g., increasing reliance on energy-dense foods) [14,15,16,17,18] and aligns with evidence that food insecurity and constrained household resources are associated with poorer dietary quality [13,36]. It is also consistent with household production perspectives, in which material resources relax constraints on food choice and enable households to convert inputs into health-related outputs, such as nutritionally balanced meals and sustained health practices [23,24]. This interpretation is further supported by evidence that intergenerational co-residence is associated with reduced poverty risk among single mothers in Japan [11,32]. However, poverty rates remain high even after accounting for co-residence, underscoring persistent structural disadvantage.

Second, shared dinner frequency was behaviorally meaningful but did not differentiate groups. Neither SingleP1 nor SingleP2 was significantly associated with shared dinner frequency after adjusting for covariates, yet shared dinners were positively associated with several dietary outcomes (dietary balance, breakfast consumption, healthy awareness, and slow eating). This finding suggests that, within this sample, single mothers and partnered mothers did not differ in how often they shared dinners, even though shared meals remained a marker of healthier patterns—consistent with previous evidence linking family meals to improved dietary quality and broader well-being outcomes [21,22]. A plausible explanation is limited between-group variation in shared dinner frequency, as most respondents reported having dinner with family almost every day (81.58%), suggesting a possible ceiling effect.

Third, time affluence did not materially contribute as a mediator in the model comparisons. This finding suggests that perceived time availability is not the operative constraint unless it is translated into stable daily routines. In this sense, shared dinner frequency functions as a more behaviorally diagnostic indicator of routine execution capacity, consistent with theoretical accounts of time allocation [23] and empirical findings that structured shared meals support healthier dietary behaviors [21,22]. The stronger association of breakfast consumption with shared dinner frequency rather than economic status also aligns with Japanese evidence that breakfast skipping is associated with poorer dietary quality and adverse lifestyle factors [37], highlighting the importance of routine coordination rather than purchasing power alone.

The cross-group comparison further clarifies why intergenerational co-residence was associated with weaker economic disadvantage. The smaller negative association for SingleP2 is consistent with intergenerational support mechanisms that reduce the effective economic burden on single mothers through income pooling and shared living costs [32,33]. For SingleP2, indirect effects via economic status were also observed for several outcomes; however, only healthy dietary awareness retained a significant total effect. This pattern indicates that economic disadvantage was detectable in both single-mother subgroups but translated into broader and more consistent overall vulnerability only among SingleP1. Evidence from Japanese national surveys indicates that co-residence can shape the measured poverty risk of single mothers [11] and contribute to economic well-being, supporting this interpretation. However, SingleP2 remained negatively associated with economic status relative to partnered mothers, underscoring that family support cannot fully offset the structural disadvantages of single motherhood—a pattern consistent with broader research on gendered poverty risk [25]. Thus, reliance on private family buffering is insufficient, and public support remains necessary.

Beyond mediation, SingleP1 showed direct disadvantages in dietary balance, breakfast consumption, and healthy dietary awareness that remained after accounting for economic status and shared dinner frequency. This residual pattern implies that a poverty-only explanation is insufficient. Table 4 supports this interpretation by showing that economic disadvantage accounted for most of the association with healthy dietary practices, accounted for part of the association with dietary balance and healthy dietary awareness, and did not account for the association with breakfast consumption. Additional pathways are likely to contribute, including psychological stress, caregiving burden, and irregular employment conditions [20,26,30,31]. Work schedules, job strain, and job type may also shape meal regularity, fatigue, and opportunities for home food preparation [30], but these factors were not available in the survey. Prior research has shown that single mothers are more likely than partnered mothers to experience depression, anxiety, and parenting stress [28], and Japanese single mothers without intergenerational co-residence are particularly vulnerable to psychological distress, social isolation, and adverse health-related coping behaviors such as smoking and alcohol use [29]. Chronic stress has been associated with unhealthy eating behaviors and reward-driven food choices [20,31]. Moreover, food insecurity has been shown to be associated with dietary vulnerability among Japanese households [36]. The absence of significant direct associations for SingleP2 across the five outcomes further suggests that living with grandparents may mitigate some of these noneconomic mechanisms (e.g., childcare assistance, household labor sharing, and emotional support) [32,33], even if such buffering is not directly observable through the shared dinner frequency measure. Taken together, this subgroup contrast supports a resource-distribution perspective on family structure: intergenerational co-residence does not merely change household size; it alters the underlying capacity to convert time and financial resources into diet-related behaviors. The most vulnerable group comprises single mothers without access to intergenerational support.

Control-variable patterns place these mechanisms within a broader context. Older age was associated with more favorable dietary balance and health-oriented practices and awareness, consistent with evidence that younger Japanese adults exhibit less healthy eating patterns (e.g., higher rates of breakfast skipping and less balanced diets) than older generations [37]. In contrast, age was negatively associated with shared dinner frequency, plausibly reflecting life-course differences in schedule synchronization. Residence in an ordinance-designated city was positively associated with economic status, suggesting spatial heterogeneity in perceived household finances. Survey-year indicators showed a notable divergence: shared dinner frequency increased in later years, whereas economic status worsened. This pattern is compatible with documented inflationary pressures in Japan, particularly food price increases captured in consumer price index statistics [38], and aligns with evidence that the COVID-19 pandemic affected dietary practices and food security among Japanese adults [5,6,7]. These findings highlight that more frequent shared meals under shock conditions do not necessarily imply improved diet quality when purchasing power erodes.

These findings identify a clear priority group and suggest pathway-targeted interventions. SingleP1 mothers should be treated as a focal vulnerable population because their disadvantages are greatest and persist partly beyond the measured mediators. Policy design should therefore address both resource capacity (the dominant economic pathway) and routine execution capacity (the pathway linked to shared dinner frequency). Feasible measures include targeted benefit supplements and relief for essential costs (food and housing) with simplified access, alongside time- and routine-related supports such as expanded affordable childcare (extended hours and after-school care), schedule stability protections, and community-based meal support programs (school-linked meals and subsidized community dining). In Japan, child-rearing and single-parent support are implemented through a combination of national schemes and municipality-specific programs, and the content of self-support measures differs across local governments [39]. Existing child-related cash benefits are primarily structured by the age of the child and standard eligibility rules, with additional provisions for the third and subsequent children, rather than being explicitly weighted by the vulnerability of single-mother households [40]. A more targeted approach may therefore be to add supplementary support for vulnerable single-mother households, particularly those without intergenerational co-residence, and to link such support to existing municipal *Shokuiku* channels [41]. Interventions that support routine formation and shared meal practices may improve dietary outcomes [21,22]. Empirical research in Europe indicates that stronger public welfare systems are associated with greater parental financial support and caregiving assistance for adult children [42]. Within the *Shokuiku* framework, the evidence supports shifting from information-only approaches to constraint-sensitive, low-burden skills support (e.g., rapid balanced meal routines, low-cost menu planning under price pressure, and decision-load reduction), delivered through schools, municipal public health services, and maternal–child health channels.

This study has several limitations that should temper causal interpretation and guide future work. First, the design is repeated cross-sectional; although the path framework is theory-driven, temporal ordering cannot be fully established, and reverse causality (e.g., healthier routines influencing perceived economic strain) cannot be excluded. As an observational repeated cross-sectional study, the analysis also remains vulnerable to residual confounding from unmeasured individual, household, and contextual factors. Second, key constructs rely on self-reported Likert-type items, which may be subject to reporting bias and measurement non-equivalence across survey modes (in-person in 2018–2019 vs. mail and web-based surveys in 2020–2022). Third, the analytic sample includes a relatively small number of single mothers—particularly SingleP2—limiting statistical power to detect modest subgroup effects and increasing uncertainty around non-significant paths or marginally significant paths. Accordingly, finding for SingleP2 should be interpreted with caution, as some substantively meaningful associations may not have reached conventional significance thresholds because of limited power. Fourth, the set of mediators was necessarily incomplete: the survey lacks validated measures of psychological distress and food insecurity modules, although such constructs are strongly associated with diet quality [13,36] and stress-related eating [20,31]. Residual confounding due to these unavailable variables therefore cannot be ruled out. Future longitudinal research should integrate dynamic transitions into single motherhood, employment instability [30], and changes in household economic conditions to more fully test causal pathways.

Future research could strengthen causal inference and expand the theoretical scope in several directions. Longitudinal designs (panel or cohort data) are needed to examine dynamic transitions into single motherhood, changes in economic conditions, and subsequent dietary trajectories. More granular measures of food insecurity (e.g., experience-based modules), time scarcity (working hours, commuting time, and childcare time), and mental health would enable explicit modeling of stress–buffering and time-allocation mechanisms alongside economic pathways. Given potential mode effects across the pandemic period, future studies should test measurement invariance across years and survey modes or triangulate findings with objective dietary records (e.g., national nutrition surveys) and administrative indicators. Finally, heterogeneity within single-mother households warrants closer attention: differences by employment type, number and age of children, receipt of child support, and local policy environments may reveal which policy levers most effectively narrow dietary disparities.

## 5. Conclusions

In conclusion, this study examined whether economic disadvantage explains dietary vulnerability among single mothers in Japan and whether intergenerational co-residence differentiates these pathways. The findings showed that economic disadvantage accounted for a substantial share of dietary vulnerability among single mothers in Japan, but not all of it. The clearest and most consistent disadvantages were observed among single mothers not co-residing with parents, whereas those co-residing with parents showed a weaker and more selective pattern of vulnerability. These findings indicate that intergenerational co-residence was associated with reduced overall disadvantage, although it did not eliminate economic hardship. By empirically testing mediation and distinguishing single mothers by co-residential status, this study helps fill a gap in the literature, which has often treated economic status and family structure as parallel predictors and single mothers as a homogeneous group. Future research should incorporate longitudinal designs and richer measures of food insecurity, time scarcity, and psychological stress to identify additional mechanisms and refine intervention targets. Policies should simultaneously strengthen single mothers’ resource capacity (e.g., targeted income and essential-cost subsidies) and time- and routine-related capacity (e.g., expanded childcare, schedule stability protections, and low-burden *Shokuiku* combined with community meal support programs) to reinforce daily healthy eating practices.

## Figures and Tables

**Table 1:** Respondents’ Characteristics.

Variable	Mean	SD
Age (20–55)	41.37	6.86
Economic status (five-point scale)	3.13	0.99
Time affluence (five-point scale)	2.72	1.12
Variable	Frequency	%
Single motherhood		
Living with grandparents	38	2.88
Living without grandparents	72	5.46
Partnered mothers	1209	91.66
Frequency of having dinner with family		
Rarely	19	1.44
1 day per week	25	1.90
2–3 days per week	93	7.05
4–5 days per week	106	8.04
Almost every day	1076	81.58
Ordinance-designated city		
Yes	398	30.17
No	921	69.83
Survey year		
2018	249	18.88
2019	230	17.44
2020	273	20.70
2021	292	22.14
2022	275	20.85

Note: Authors’ calculations.

**Table 2:** Model fit and nested comparisons.

Model	χ^2^	df	Δχ^2^	Δdf	*p* (Δχ^2^)	CFI	TLI	RMSEA	SRMR
Full model	7.06	5	—	—	—	0.999	0.992	0.018	0.001
Model without time affluence	12.60	10	5.32	5	0.378	0.998	0.995	0.009	0.014
Model without shared dinner frequency	53.00	10	46.00	5	<0.001 ***	0.971	0.920	0.045	0.057
Model without economic status	66.30	10	67.20	5	<0.001 ***	0.962	0.895	0.035	0.065

Note: Scaled *χ*^2^ difference tests are reported relative to the full model. *** *p* < 0.001.

**Table 3:** Results of path analysis with standardized estimates.

Outcome	Predictor	Std. Est.	SE	Z	*p*
Dietary balance	Age	0.10	0.03	3.20	0.001
	SingleP1	−0.13	0.03	−4.28	<0.001
	SingleP2	0.02	0.03	0.72	0.474
	2020	−0.15	0.03	−4.20	<0.001
	2021	−0.16	0.03	−4.88	<0.001
	2022	−0.18	0.03	−5.35	<0.001
	Economic status	0.11	0.03	3.61	<0.001
	Shared dinner frequency	0.14	0.04	3.32	0.001
Breakfast consumption	Age	0.06	0.04	1.59	0.113
	SingleP1	−0.15	0.03	−4.57	<0.001
	SingleP2	−0.03	0.04	−0.69	0.490
	2020	−0.07	0.05	−1.48	0.139
	2021	−0.07	0.05	−1.59	0.111
	2022	−0.08	0.04	−1.73	0.083
	Economic status	0.04	0.04	1.08	0.280
	Shared dinner frequency	0.18	0.05	3.45	0.001
Healthy awareness	Age	0.10	0.03	3.14	0.002
	SingleP1	−0.09	0.03	−3.40	0.001
	SingleP2	−0.06	0.03	−1.91	0.056
	2020	−0.15	0.03	−4.18	<0.001
	2021	−0.10	0.04	−2.70	0.007
	2022	−0.04	0.04	−1.10	0.273
	Economic status	0.20	0.03	6.49	<0.001
	Shared dinner frequency	0.19	0.04	4.45	<0.001
Healthy dietary practices	Age	0.11	0.03	3.60	<0.001
	SingleP1	−0.04	0.03	−1.30	0.193
	SingleP2	0.01	0.03	0.39	0.699
	2020	−0.10	0.04	−2.72	0.007
	2021	−0.02	0.03	−0.74	0.457
	2022	−0.07	0.04	−1.82	0.069
	Economic status	0.19	0.03	6.15	<0.001
	Shared dinner frequency	0.05	0.04	1.13	0.257
Slow eating	Age	0.02	0.03	0.53	0.597
	SingleP1	0.04	0.03	1.23	0.219
	SingleP2	0.05	0.03	1.88	0.060
	2020	−0.11	0.03	−3.12	0.002
	2021	−0.09	0.03	−2.50	0.012
	2022	−0.07	0.03	−2.05	0.041
	Economic status	0.14	0.03	4.71	<0.001
	Shared dinner frequency	0.12	0.04	2.99	0.003
Economic status	Age	−0.05	0.03	−1.82	0.069
	SingleP1	−0.18	0.03	−5.75	<0.001
	SingleP2	−0.09	0.03	−3.31	0.001
	2020	−0.02	0.03	−0.55	0.582
	2021	−0.07	0.03	−2.19	0.029
	2022	−0.09	0.03	−2.69	0.007
	Ordinance-designated city	0.07	0.03	2.47	0.013
Shared dinner frequency	Age	−0.16	0.04	−4.20	<0.001
	SingleP1	−0.05	0.04	−1.47	0.142
	SingleP2	−0.01	0.04	−0.38	0.702
	2020	0.15	0.05	3.29	0.001
	2021	0.09	0.04	2.15	0.032
	2022	0.13	0.04	2.90	0.004
	Ordinance-designated city	0.01	0.04	0.12	0.902

Note: Authors’ calculations.

**Table 4:** Standardized effect decomposition from single parenthood to dietary outcomes.

Path	Direct		Indirect (via Econ)		Total		Interpretation by Economic Disadvantage
	Std. Est.	SE	Std. Est.	SE	Std. Est.	SE	
SingleP1 → Dietary balance	−0.126 ***	0.029	−0.019 **	0.006	−0.146 ***	0.028	Partially explained
SingleP1 → Breakfast consumption	−0.148 ***	0.032	−0.008	0.007	−0.155 ***	0.031	Not explained
SingleP1 → Healthy awareness	−0.094 ***	0.027	−0.035 ***	0.008	−0.129 ***	0.027	Partially explained
SingleP1 → Healthy dietary practices	−0.036	0.028	−0.033 ***	0.008	−0.069 *	0.027	Mainly explained
SingleP1 → Slow eating	0.037	0.030	−0.026 ***	0.007	0.011	0.030	No vulnerability
SingleP2 → Dietary balance	0.022	0.030	−0.009 *	0.004	0.012	0.030	No vulnerability
SingleP2 → Breakfast consumption	−0.025	0.036	−0.004	0.004	−0.029	0.036	No vulnerability
SingleP2 →Healthy awareness	−0.064	0.033	−0.017 **	0.006	−0.081 *	0.033	Partially explained
SingleP2 →Healthy dietary practices	0.011	0.028	−0.016 **	0.005	−0.005	0.029	No vulnerability
SingleP2 → slow eating	0.053	0.028	−0.012 **	0.005	0.041	0.028	No vulnerability

Note: Authors’ own calculations. * *p* < 0.05, ** *p* < 0.01, *** *p* < 0.001.

## Data Availability

Individual-level data from the questionnaire surveys are publicly available upon request from the Japan Social Science Data Archive, Center for Social Research and Data Archives, Institute of Social Science, The University of Tokyo.

## Abbreviations

The following abbreviations are used in this manuscript:

WLSMV	Weighted Least Squares Mean and Variance Adjusted

## Appendix A

**Table A1:** Polychoric correlations and multicollinearity diagnostics among key variables.

	Economic Status	Time	Shared Dinner Frequency	Balance	Breakfast	Healthy Awareness	Healthy Diet Practices	Slow Eating
Economic status	1.000	0.400	0.117	0.153	0.100	0.236	0.191	0.152
Time	0.400	1.000	0.080	0.096	0.087	0.114	0.120	0.116
Shared dinner frequency	0.117	0.080	1.000	0.119	0.178	0.185	0.035	0.118
Dietary balance	0.153	0.096	0.119	1.000	0.281	0.425	0.288	0.048
Breakfast consumption	0.100	0.087	0.178	0.281	1.000	0.267	0.195	0.123
Healthy awareness	0.236	0.114	0.185	0.425	0.267	1.000	0.555	0.185
Healthy dietary practices	0.191	0.12	0.035	0.288	0.195	0.555	1.000	0.219
Slow eating	0.152	0.116	0.118	0.048	0.123	0.185	0.219	1.000

Variance Inflation Factor (VIF) diagnostics for mediators: time = 1.002; shared dinner frequency = 1.002.
